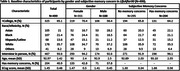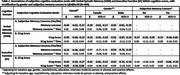# Gender Differences in the Association Between Subjective and Objective Cognitive Decline in a Diverse Cohort: *LifeAfter90* Study

**DOI:** 10.1002/alz.091848

**Published:** 2025-01-03

**Authors:** Nancy X Chen, María M. M. Corrada, Paola Gilsanz, Sarah Tomaszewski Farias, Kristen M. George, Alexander Ivan B. Posis, Claire C. Meunier, Yi Lor, Rachel A. Whitmer

**Affiliations:** ^1^ University of California, Davis, Davis, CA USA; ^2^ University of California, Irvine, Irvine, CA USA; ^3^ Kaiser Permanente Northern California Division of Research, Oakland, CA USA; ^4^ University of California, Davis, Sacramento, CA USA

## Abstract

**Background:**

Few studies have investigated the relationship between subjective cognitive concerns and objective cognitive decline in the oldest‐old or examined gender differences. We evaluated this association and stratification by gender in a diverse cohort of adults ages 90+.

**Methods:**

*LifeAfter90* is an ongoing cohort of adults ages 90+. Verbal episodic memory (VEM) and executive function (EF) were assessed every 6‐months since 2018 using the Spanish and English Neuropsychological Assessment Scales. We operationalized subjective cognition two ways: 1) Dichotomously as yes/no via self‐report of subjective memory concerns (SMC)‐ “are you concerned that you have a memory or other thinking problem?”; 2) Continuously as degree of self‐rated changes in cognition using the 16‐item Everyday Cognition Scale (ECog). Linear mixed models with random intercepts and slopes estimated associations of SMC or ECog with VEM and EF at baseline and longitudinally, pooled and stratified by gender (female/male). Models adjusted for baseline age, gender (in pooled models), race/ethnicity, education, interview mode (in‐person or phone), and practice effects. Sensitivity models further stratified by SMC.

**Results:**

Participants’ (N = 499) mean baseline age 93±2.4 years, mean follow‐up time 2.7±1.1 years, 62% women, 64% racial/ethnic minorities, 41% endorsed SMC, and mean ECog score 1.45±0.5. Overall, SMC and ECog were associated with worse baseline VEM (β_SMC_ = ‐0.44,95%CI ‐0.61,‐0.27; β_ECog_ = ‐0.40,95%CI ‐0.58,‐0.22) and worse baseline EF (β_SMC_ = ‐0.23,95%CI ‐0.39,‐0.07; β_ECog_ = ‐0.43,95%CI ‐0.59,‐0.27) (Table 2). ECog was associated with greater decline in EF (β = ‐0.05, 95%CI ‐0.10,0.00; Table 2). There were no gender differences in associations between SMC (p = 0.44) or ECog (p = 0.33) and VEM, nor ECog and EF (p = 0.76). The association between SMC and EF differed across genders (p = 0.01). In gender‐stratified models, both genders with SMC had worse baseline VEM and worse baseline EF, though not significantly for women (β_men_ = ‐0.54,95%CI ‐0.79,‐0.29; β_women_ = ‐0.07,95%CI ‐0.27,0.13) (Table 2). Both genders with greater ECog had worse baseline VEM (β_men_ = ‐0.56,95%CI ‐0.87,‐0.26; β_women_ = ‐0.33,95%CI ‐0.56,‐0.10) and worse baseline EF (β_men_ = ‐0.43,95%CI ‐0.70,‐0.14; β_women_ = ‐0.41,95%CI ‐0.61,‐0.22) (Table 2). After stratification by SMC, among those with SMC, ECog was associated with worse baseline EF (β = ‐0.47,95%CI ‐0.68,‐0.26; Table 2).

**Conclusion:**

In this diverse cohort of 90+, the association between subjective memory concerns and worse baseline EF was more pronounced in men than in women.